# Jejunal Diverticulosis Presenting With Small Bowel Obstruction: A Diagnostic Challenge

**DOI:** 10.7759/cureus.56205

**Published:** 2024-03-15

**Authors:** Martha Gismondi, Omar H Ali, Omotayo Ajao, Jamasp Dastur

**Affiliations:** 1 Colorectal Surgery, Norfolk and Norwich University Hospital National Health Service (NHS) Foundation Trust, Norwich, GBR

**Keywords:** general surgery, enterolith, small bowel enterotomy, small bowel obstruction, jejunal diverticulosis

## Abstract

We report the case of a woman presenting with small bowel obstruction secondary to an enterolith that formed within a jejunal diverticulum. Prior to this acute presentation, the patient had experienced regular abdominal pain albeit not as severe as the current episode. The CT scan on admission required review by two consultant radiologists before the cause of the small bowel obstruction was diagnosed. Successful surgical management was performed involving a laparotomy, small bowel enterotomy, and removal of the enterolith. Although complications secondary to jejunal diverticula are documented, there is minimal literature on the complexities of making the diagnosis and the best management approach that should be adopted.

## Introduction

We report the case of a patient who presented with a small bowel obstruction secondary to an enterolith that formed within a jejunal diverticulum. Jejunal diverticula are outpouchings of the small intestine occurring at weak points in the bowel wall with an incidence of 0.3%-2.5% [1]. They are mostly discovered incidentally either as a result of an unrelated pathology [1] or due to an associated complication such as perforation, haemorrhage, or bowel obstruction, as in our reported case. This article would like to highlight not only the management we adopted for this rare condition but also the complexity of making the diagnosis.

## Case presentation

A 67-year-old woman presented to our hospital with a seven-day history of colicky abdominal pain associated with nausea and normal bowel movement. The patient described that she had regularly experienced similar pain around once every six months before this admission, albeit not as severe as the current episode.

**Figure 1:**
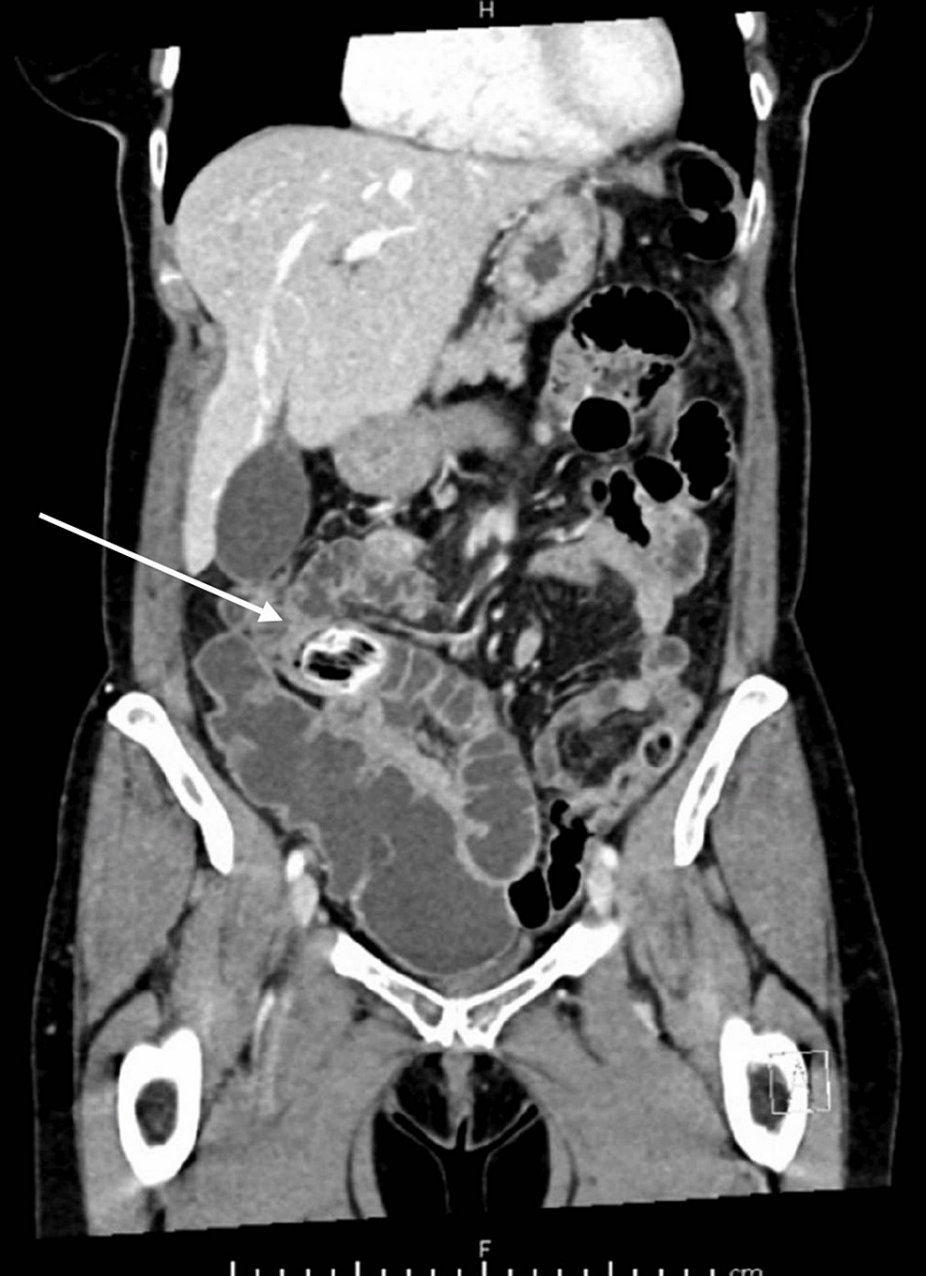
Coronal view of CT AP CT AP: Computed tomography scan of the abdomen and pelvis The image shows the presence of a jejunal diverticulum and a radio opacity, likely to represent an enterolith (white arrow).

On initial examination, right iliac fossa tenderness was elicited without clinical peritonism. Her inflammatory markers were raised: C-reactive protein (CRP) 106 mg/L and white cell count (WCC) 11.1 x 10^9^/L. An initial computed tomography scan of the abdomen and pelvis (CT AP) (Figure 1) was originally reported as the presence of a small volume of pelvic free fluid but no enhancing collection or abscess, a possible small volume of intraperitoneal free air with peritoneal enhancement suggestive of peritonitis, and dilated small bowel loops secondary to paralytic ileus. The CT AP was later reviewed by two consultant radiologists. The first addendum to the CT report described the presence of a strange-appearing structure within the lumen of a distended small bowel loop within the pelvis that had a markedly radio-opaque surface and radio-lucent centre, which could represent either a bezoar or a stercolith. Although its position inside the small bowel would be surprising in the latter case, it could give rise to intermittent obstruction. The second consultant radiologist reported the presence of a jejunal diverticula and a radio-opacity, which was likely to represent an enterolith.

**Figure 2:**
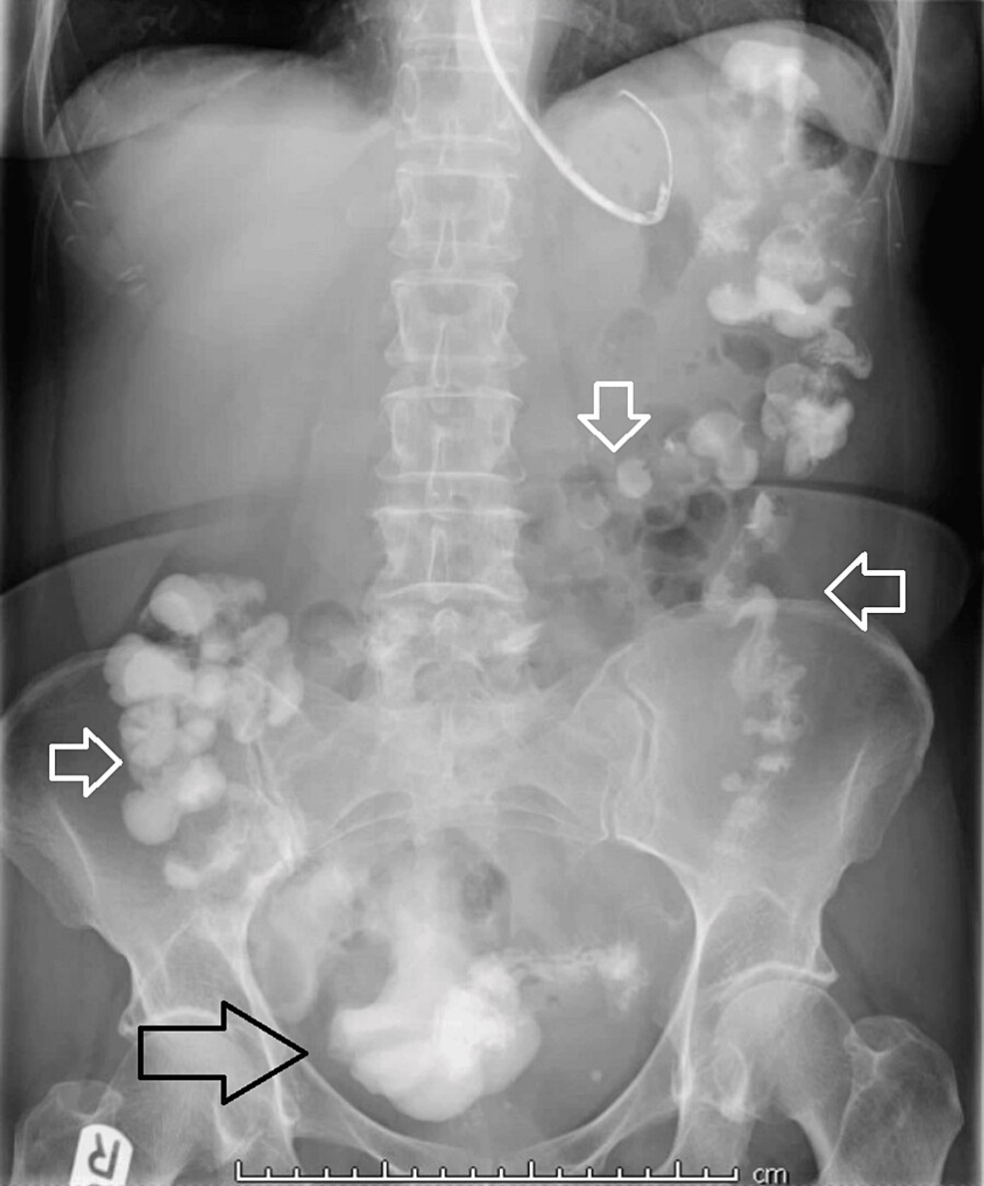
Plain abdominal film performed 24 hours after the contrast administration The plain abdominal film shows the successful flow of gastrografin throughout the large bowel, passing beyond the level of obstruction caused by the enterolith in the jejunal diverticulum. The black arrow indicates evident contrast visualisation in the rectum, while the white arrows demonstrate the presence of contrast throughout the colon.

Initial management consisted of empirical antibiotics, nasogastric tube insertion, and administration of gastrografin with the view of surgical intervention if conservative management fails. Gastrografin passed successfully through the bowel as can be seen on the plain abdominal film, performed 24 hours after the contrast administration (Figure 2). This resulted in the resolution of the patient's symptoms and facilitated her initial discharge.

**Figure 3:**
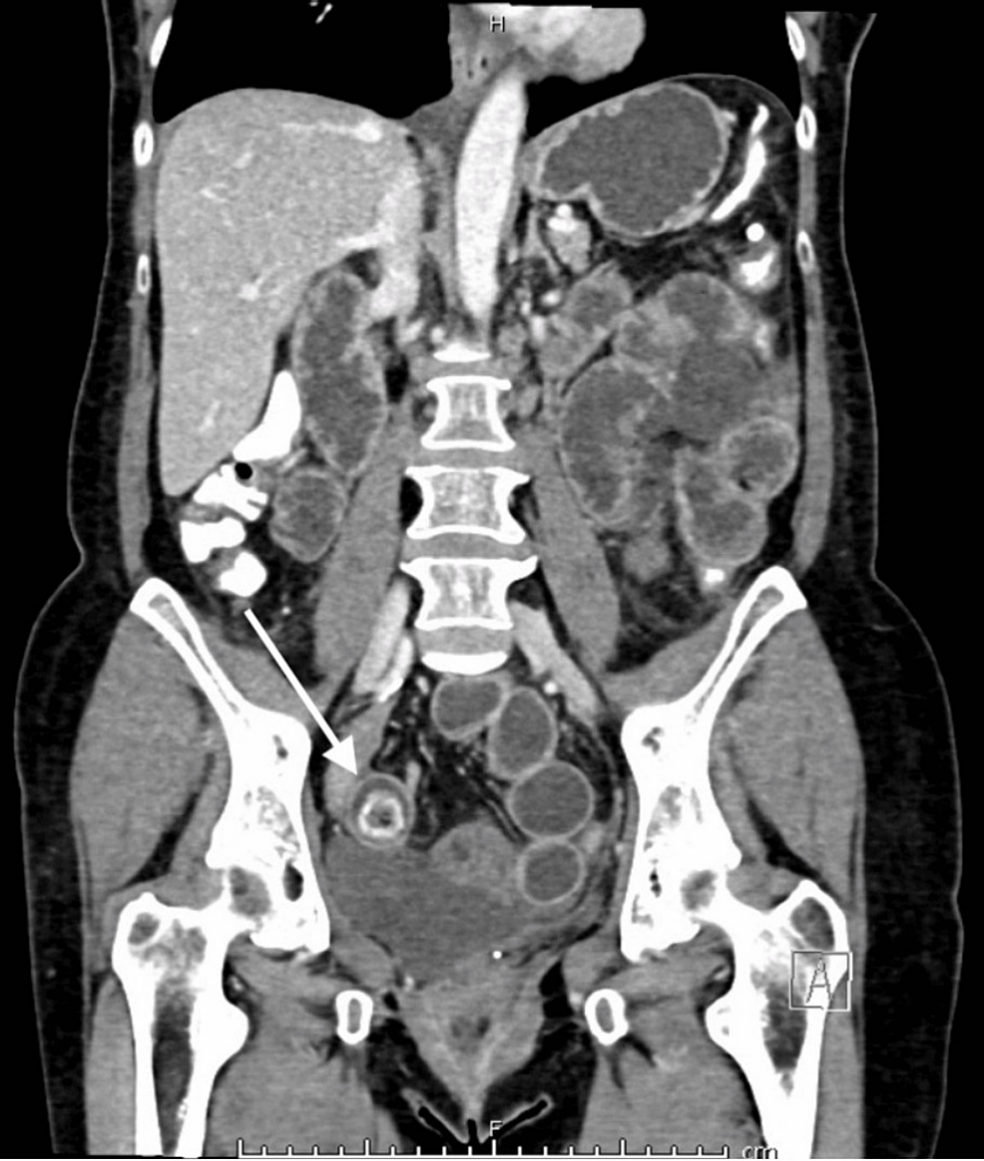
Repeat CT AP during the patient’s second admission CT AP: Computed tomography scan of the abdomen and pelvis This coronal view of CT AP demonstrates high-grade small bowel obstruction secondary to an enterolith (white arrow), which has migrated to the distal ileum from a jejunal diverticulum.

**Figure 4:**
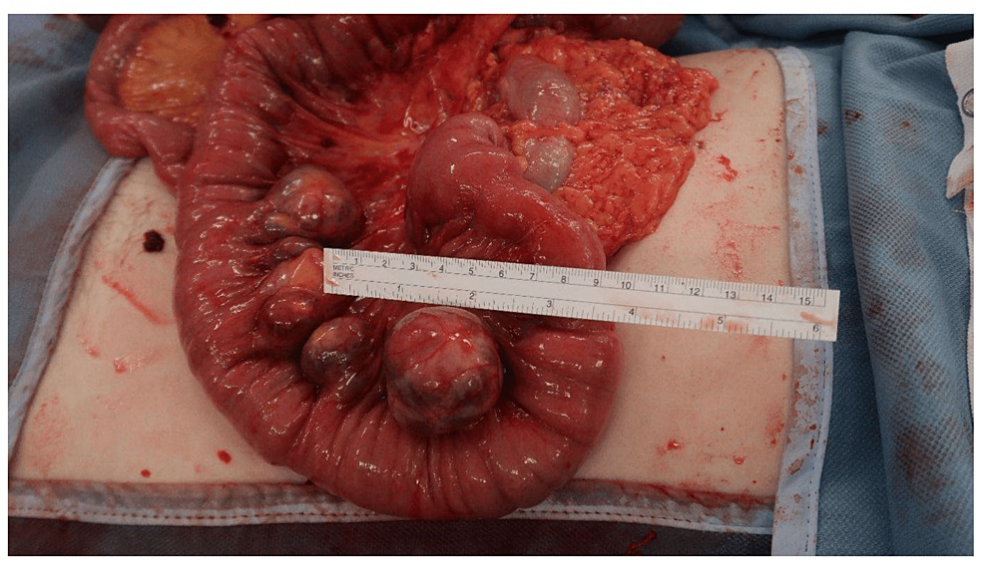
Intraoperative image Intraoperative view of jejunal diverticula on the mesenteric border found at 45 cm from the duodenojejunal flexure.

The patient re-presented a day later with worsening generalised colicky abdominal pain associated with nausea and vomiting and no bowel motion over the preceding 24 hours. A repeat CT AP showed the presence of high-grade small bowel obstruction secondary to a presumed enterolith that had migrated to the distal ileum from a jejunal diverticulum (Figure 3). Therefore, she underwent a laparotomy with small bowel enterotomy and removal of the enterolith. Intraoperatively, the small bowel was examined from the duodeno-jejunal flexure downstream. Adhesions between the jejunum and the caecum were carefully dissected. Jejunal diverticula were found at 45 cm from the duodenojejunal flexure (Figure 4). A small bowel serosal tear was found at 60 cm from the duodenojejunal flexure and repaired with 3/0 polydioxanone suture (PDS) interrupted sutures. The enterotomy was performed at 210 cm from the duodeno-jejunal flexure and the enterolith was removed. This was followed by a strictureplasty. After intra-abdominal washout, haemostasis was achieved.

Four days postoperatively, the patient reported right iliac fossa tenderness and failed to open her bowels. Her WCC was within normal limits, but her CRP was markedly elevated (340 mg/L). Therefore, an urgent CT AP was requested to rule out any collection, antibiotics were started, and total parenteral nutrition was initiated due to the prolonged period of starvation. The scan reported the presence of paralytic ileus as well as collections in the lower abdomen and the presacral region. After discussing the case with the interventional radiology (IR) team, a decision was made to undertake IR drainage two days following her latest CT AP. 400 ml of serosanguinous fluid was aspirated, likely to represent a haematoma. Two weeks later, the drain output was minimal, her paralytic ileus resolved, inflammatory markers were unremarkable, and she had normal bowel function. Subsequently, the patient was discharged.

## Discussion

Small bowel diverticula can be either congenital (e.g. Meckel’s diverticulum) or acquired. Usually, acquired diverticula arise on the mesenteric border (Figure 4) rather than the antimesenteric border as in congenital cases [2]. The pathophysiology of acquired small bowel diverticula entails intestinal dysmotility, which produces high intraluminal pressure, and weak points found in the muscular layer of the bowel wall secondary to penetration of feeding arteries [3]. Most small bowel diverticula are found in the duodenum whilst the incidence of jejunal and ileal diverticula has been reported at only 18% [1].

Jejunal diverticula are usually asymptomatic until complications arise as in our case report. Several complications have been reported in the literature including strangulation [4], volvulus [5], perforation [6], diverticulitis, and small bowel obstruction. Overall, complications have been reported in around 10% of patients with jejunal diverticula [3]. 

In our case, recurrent non-severe abdominal pain was experienced by the patient before the diagnosis of jejunal diverticula. We believe that this could be due to either mild episodes of diverticulitis or growth of the enterolith inside the diverticulum until it reached a considerable size to cause small bowel obstruction. For instance, enteroliths are usually associated with the development of small bowel obstruction when their size is > 2-3 cm [7].

Enteroliths usually consist of faecal matter, calcium phosphates, magnesium, bacteria, and unconjugated bile acids [7]. Choleic acid enteroliths usually develop in acidic environments such as the duodenum and the proximal small bowel, while calcium salt stones are mostly found in alkaline environments such as the distal small bowel [7].

Our case highlights the challenge of radiological diagnosis of jejunal diverticulosis. This is mainly due to the rarity of diverticula in this location. Jejunal diverticulosis is present on CT scans as round or ovoid sac-like protrusions with a neck arising from the bowel wall [8]. The initial CT report of our patient did not describe the presence of any small bowel wall protrusions and a diagnosis of jejunal diverticulosis was only made after a secondary review by a consultant gastrointestinal radiologist. 

This case report emphasises the need for surgical intervention, rather than conservative management, once a diagnosis of small bowel obstruction secondary to enterolith formation in a jejunal diverticulum has been made. This was also recommended by Bisheet and Mena in their 2022 case report where either an enterotomy with stone extraction or a laparotomy with propulsion of stone towards the colon was advised in the presence of bowel obstruction [9]. We opted for an enterotomy with enterolith extraction. Resection of the diverticula would not have been beneficial for the patient since this was her first presentation, and bowel resection would have increased the likelihood of complications such as an anastomotic leak.

As reported by Lee and Menezes, it is necessary to include enterolith impaction causing bowel obstruction as a differential diagnosis in patients with known small bowel diverticulosis, especially because enterolith formation can be recurrent [10]. The exact pathophysiological mechanism by which enteroliths form remains unclear. Furthermore, the incidence of recurrent enterolith formation and preventive measures in patients affected by small bowel diverticulosis remain unknown at present [10].

## Conclusions

Jejunal diverticulosis is a rare pathology that can be associated with complications such as enterolith formation, which can cause small bowel obstruction. Even with the availability of a CT scan, such a diagnosis can be challenging. We believe that in this clinical context, a second opinion from a gastrointestinal radiologist should always be sought preoperatively to plan surgical management. As depicted in our case report, conservative management is likely to fail. Hence, a surgical option should be adopted if the patient is fit enough for surgery.
